# Classifying sepsis from photoplethysmography

**DOI:** 10.1007/s13755-022-00199-3

**Published:** 2022-10-31

**Authors:** Sara Lombardi, Petri Partanen, Piergiorgio Francia, Italo Calamai, Rossella Deodati, Marco Luchini, Rosario Spina, Leonardo Bocchi

**Affiliations:** 1 Department of Information Engineering, University of Florence, Via S. Marta, 3, 50139 Florence, Italy; 2 Faculty of Information Technology and Electrical Engineering, University of Oulu, Pentti Kaiteran katu 1, 90570 Oulu, Finland; 3 S.O.C. Anestesia e Rianimazione, Ospedale S. Giuseppe, viale Giovanni Boccaccio, 16, 50053 Empoli, Italy

**Keywords:** Deep learning, Photoplethysmography, PPG, CNN, Sepsis, ICU

## Abstract

Sepsis is a life-threatening organ dysfunction. It is caused by a dysregulated immune response to an infection and is one of the leading causes of death in the intensive care unit (ICU). Early detection and treatment of sepsis can increase the survival rate of patients. The use of devices such as the photoplethysmograph could allow the early evaluation in addition to continuous monitoring of septic patients. The aim of this study was to verify the possibility of detecting sepsis in patients from whom the photoplethysmographic signal was acquired via a pulse oximeter. In this work, we developed a deep learning-based model for sepsis identification. The model takes a single input, the photoplethysmographic signal acquired by pulse oximeter, and performs a binary classification between septic and nonseptic samples. To develop the method, we used MIMIC-III database, which contains data from ICU patients. Specifically, the selected dataset includes 85 septic subjects and 101 control subjects. The PPG signals acquired from these patients were segmented, processed and used as input for the developed model with the aim of identifying sepsis. The proposed method achieved an accuracy of 76.37% with a sensitivity of 70.95% and a specificity of 81.04% on the test set. As regards the ROC curve, the Area Under Curve reached a value of 0.842. The results of this study indicate how the plethysmographic signal can be used as a warning sign for the early detection of sepsis with the aim of reducing the time for diagnosis and therapeutic intervention. Furthermore, the proposed method is suitable for integration in continuous patient monitoring.

## Introduction

The Sepsis-3 task force in 2016 defined sepsis as a life-threatening organ dysfunction caused by a dysregulated host response to an infection [1]. This condition is a major global health problem that represents a significant burden to the health care systems of different countries. Sepsis is one of the leading causes of death in intensive care unit (ICU) affecting 49 million people annually (year 2017 [2]). This feared condition occurs in up to 30% of ICU patients and result in a mortality rate twice as high as that of non-septic patients [3]. All of this indicates how the recognition and treatment of sepsis should be considered as medical emergencies in order to reduce time for treatment and risk for patients [4, 5]. This is very important because sepsis is a rapidly progressive condition and the mortality rate of patients has been shown to be correlated to timeliness of a therapeutic intervention highlighting the importance of early detection and treatment [6, 7]. In this sense, a few hours of delay in detection and treatment from the onset are associated with a reduction in survival rate [6, 8, 9]. Unfortunately, there is currently no gold-standard test for the diagnosis of sepsis. Consequently, different sepsis scoring systems (SSS) are commonly used in clinical practice. Strengths and weaknesses have been recognized for each of these sepsis screening tools, as well as areas of preferential application [5, 10]. Manually tabulated SSS such as Systemic Inflammatory Response Syndrome (SIRS) criteria [11] and Sequential Organ Failure Assessment (SOFA) [12] are usually used to identify sepsis. These tools include the evaluation of several parameters obtained from laboratory tests. Conversely, Quick-SOFA (qSOFA) is a scoring system [1] which utilizes only three independent non-laboratory test variables and is often used for a quick assessment that may require for further investigations. This tool is normally used for the purpose of predicting organ dysfunction and death in patients with or suspected sepsis in emergency department [4, 5, 7]. Unfortunately, the presence of multiple definitions of sepsis and recommendations for the use of different SSS can lead to confusion in clinical practice and hinder a quick diagnosis and treatment of sepsis as well as the definition of shared treatment protocols [4, 10, 13, 14]. Furthermore, the use of SSS may lead to costs such as those for laboratory tests and time to obtain the score in addition to showing limits regarding the sensitivity [2]. These limitations can be particularly evident in low and middle-income countries in which a timely execution of laboratory tests can be difficult. It has recently been suggested that it is useful to use multiple SSS at the same time (mixed models). This model can further hinder the timely evaluation of patients [5, 10, 15]. All this may limit the use of the SSS and indicates the need to continue studying tests and procedures that can promptly recognize the presence of sepsis. In this context, the availability of electronic clinical records together with data relating to continuous monitoring of vital signs could offer important supporting methods for the sepsis identification. Among the data available in these datasets there are those relating to microcirculation. These data are important because multiple clinical trials have shown common microcirculatory dysfunctions in sepsis patients [16, 17]. The alterations of the microcirculation have been associated with organ failure and increase in mortality [18–21]. Microcirculatory dysfunctions in sepsis patients reflect themselves on parameters that can be easily evaluated at the skin level, as the photoplethysmogram (PPG). This signal is commonly monitored using devices such as the pulsi-oximeter. This device is widely used, user-friendly and affordable. In particular, PPG is an optical signal that utilizes the absorption or reflection of the light through blood to detect changes in blood volume and oxygen saturation at a peripheral site, typically the finger. It is worth noting that the perfusion characteristics depends on the measurement site, that needs to be defined as a part of the experiment protocol [22, 23]. Photoplethysmogram is now widely used in intensive care units for cardiovascular monitoring since it allows a non-invasive, continuous and comfortable measurement. In this sense, it is important to consider that photoplethysmogram waveform contains information on heart rate, venous blood volume and peripheral vascular tone. As a whole this information can be very important because it could allow controlling the cardiovascular system.

Spectral analysis of photoplethysmogram has already been used to gain insight into the peripheral microcirculatory function of sepsis patients. Piepoli et al. [24] showed that the low-frequency (LF, 0.04–0.15 Hz) band of fingertip PPG was suppressed in septic shock patients. This is considered relevant because low-frequency band of fingertip PPG has been associated with sympathetic control over the peripheral circulation. Middleton et al. [25] reported that the mid-frequency (MF, 0.09–0.15 Hz) band of earlobe PPG had a significant decrease in power spectral density in severe stage sepsis patients, compared to controls and early stage sepsis subjects.

Traditional machine learning algorithms have previously been exploited for the detection of sepsis in ICUs. Calvert et al. [26] developed a classical machine learning algorithm to identify sepsis using many vital signs and demographic features. Other studies have subsequently further validated the same algorithm [27, 28] using different input features and different data sets. These studies showed that the algorithm outperformed standard sepsis diagnostics methods, such as tabulated scoring systems.

Mollura et al. [29] trained multiple machine learning classifiers using features extracted from continuously recorded electrocardiogram (ECG) and arterial blood pressure (ABP) signals, in order to identify sepsis within one hour of admission to ICU. The authors reported that classification results were comparable with those obtained with tabulated scores, suggesting that vital sign waveforms might be useful in the early detection of sepsis.

A lot of studies have recently used deep learning approaches to carryout medical tasks, highlighting their potential in the healthcare field [30–32]. Deep learning models automatically learn from raw data without requiring conventional feature extraction and selection steps. Among deep learning architectures, Convolutional Neural Networks (CNN) are currently the state-of-the-art technique for signal processing applications. Consequently, CNNs have been increasingly used in biomedical signal analysis [33, 34]. CNN models and photoplethysmographic signals have previously been jointly used to perform classification tasks. In this sense, some authors used spectrograms and scalograms, obtained from PPG signals, to train a CNN model to perform blood pressure classification [35, 36].

In this study, raw fingertip photoplethysmography time-series data related to ICU patients were used to train and evaluate a CNN-based model. The aim of this study was to verify the possibility of detecting sepsis in patients from the photoplethysmographic signal acquired by the pulse oximeter.

## Materials and methods

### Dataset

This study used the MIMIC-III database [37], a large, a freely accessible critical care database. MIMIC-III is provided as a collection of comma-separated value files, that we imported into a PostgreSQL relational database system. The data are organised in tables containing information such as demographics data, vital sign measurements, laboratory test results, procedures and mortality rate. The tables are linked by identifiers allowing the extraction of information on the same patient.

Waveform recordings, such as ECG and PPG, are stored in a separate database, the “MIMIC-III Waveform Database” [38]. In particular in a subset of the waveform database, the “MIMIC-III Waveform Database Matched Subset” [39], there are the recordings for which the patient has been linked to the clinical information available in the MIMIC-III database.

The MIMIC-III database contains a large heterogeneity of subjects, allowing it to be used for a variety of analytical studies. However, this heterogeneity could make the development of an efficient machine learning algorithm challenging [40]. Moreover, diagnosis are reported only as an ICD-code generated at the end of the hospitalization, without providing any information on the date of the diagnosis. Thus, we selected a subset of the subjects, identified as “sepsis” (cases) and “non-sepsis” (controls) patients. The criteria used to select sepsis and non-sepsis subjects are reported in Table 1. In this phase a custom Structure Query Language (SQL) query was used.

**Table 1:** Criteria for the definition of septic patients and control (non-septic) patients

Selection criteria for control patients	
• No death in the hospital	
• Single ICU hospitalisation	
• Single hospital admission	
• One or more following diagnosis codes (ICD-9): 311 (Depressive disorder NEC), 3051 (Tobacco use disorder), 30,000 (Anxiety state NOS), 2948 (Other persistent mental disorders due to conditions classified elsewhere), 3004 (Dysthymic disorder)	
• No sepsis diagnoses	
• Subject was present in the Matched Subset of MIMIC-III Waveform Database	

Selection criteria for sepsis patients	
• Death in the hospital	
• Single ICU hospitalisation	
• Single hospital admission	
• One or more following ICD-9 diagnosis codes: 99,591 (Sepsis), 99,592 (Severe sepsis), 78,552 (Septic shock)	
• Subject was present in the Matched Subset of MIMIC-III Waveform Database	

The selection criteria resulted in a large number of control subjects in comparison to the group of patients with sepsis. Therefore, we limited the control group to 40 subjects per ICD-9 code in order to have a more balanced selection. As a result, the group of patients with sepsis was of 178 subjects while the control group was of 200 subjects.

The MIMIC-III Waveform Database contains a variety of signals (such as ECG, ABP, PPG) but not all of them are available for each patient. Therefore, we further restricted the selection to those patients for whom the PPG signal was available. As a result the group of patients with sepsis was reduced to 147 subjects while the control group consisted of 155 subjects.

### Preprocessing

We downloaded the recordings from the Matched Subset of MIMIC-III Waveform database and extracted the PPG of selected patients using WFDB Python package [41]. Selected signals were split into 2-min segments, and segments less than 2 min were discarded. Furthermore, in order to reduce the degree of similarity within the collected signals, we kept only every other segment.

Afterwards, the regularity and quality of each 2 min sample was assessed using a template matching approach, a technique already used by other authors [42, 43]. This quality estimation was carried out using 3-s running window over the 2 min segment. We classified each window by comparing the signal acquired from the patient to an optimal template PPG signal. The similarity between the two time-series was calculated with Pearson’s correlation coefficient.

**Fig. 1:**
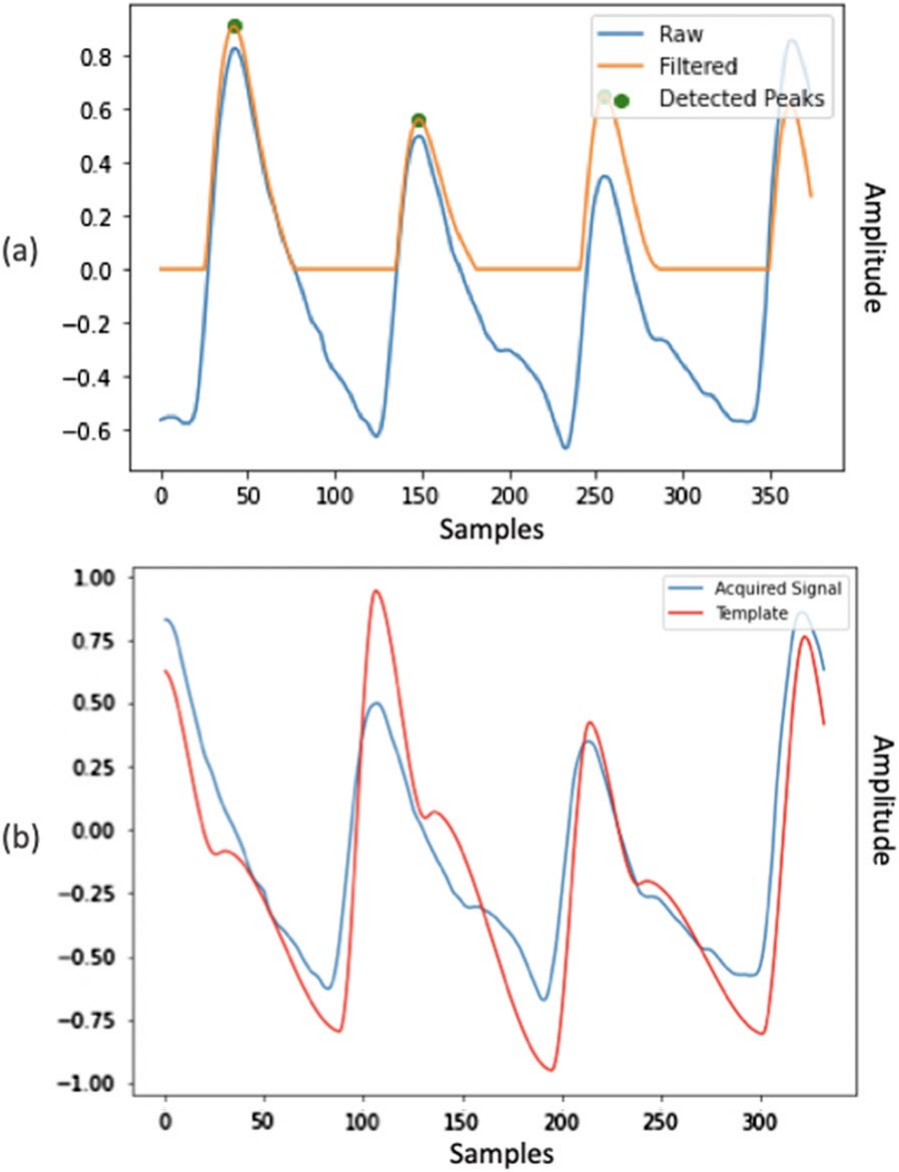
Template matching method. **a** The raw and filtered signal. The filtered signal was used to identify the position of the systolic peaks indicated by green dots. **b** Alignment between the acquired signal and the template on the first systolic peak, which allowed us to calculate the correlation coefficient between the two waveform

The template was generated using NeuroKit2 python toolbox, a package for neurophysiological signal processing [44]. The reference PPG signal was simulated without noise and motion artifacts. The simulation algorithm also requires as input the sampling frequency of the signal and the mean heart rate within each window. Sampling frequency was set to 125 Hz, which is the sampling frequency of all the signals in the waveform database. Mean heart rate was calculated considering the distance between the systolic cardiac peaks. To identify the peak locations, we first filtered the signal and then we used NeuroKit’s peak finding method, as illustrated in Fig. 1a.

**Fig. 2:**
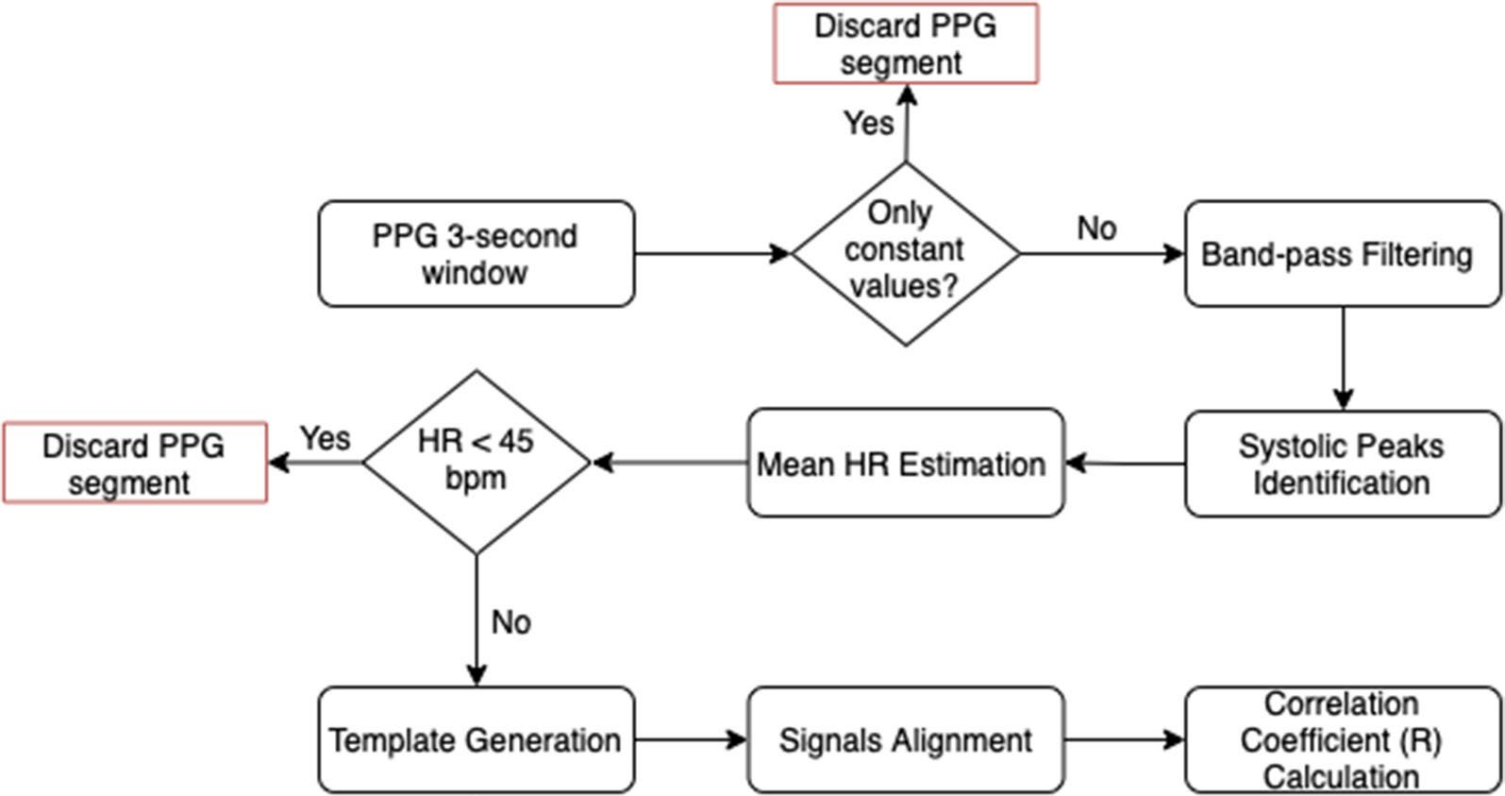
Flow chart of the template matching algorithm. The template matching procedure was performed on 3-s windows obtained from each 2-min PPG segment. The correlation coefficient values obtained for each window were stored and subsequently used to classify the quality of the 2-min sample as acceptable or unacceptable

Signal filtering was carried out using a third-order Butterworth bandpass filter with cut-off frequencies of 0.5 and 8 Hz. The objective of the signal filtering was to remove the baseline component and frequencies that are not relevant for systolic peaks. This peak finding function implements a method previously proposed by Elgendi et al. [45] based on event-related moving averages with dynamic thresholds. On the bases of the procedure reported above, we were able to identify the location of systolic peaks and therefore estimate the mean heart rate within the window considered. At this stage, we excluded segments containing windows with only constant values, for which identification of systolic peaks was not possible, and windows with an estimated mean heart rate below 45 bpm. Once the reference signal was generated, we aligned the patient-acquired window and the template signal on the first systolic peak, Fig. 1b. Hence, we calculated the Pearson correlation coefficient in order to evaluate the similarity between the two signals. A flow chart that summarises the developed template matching algorithm is shown in Fig. 2.

The 3-s windows were grouped into four classes using the thresholds for correlation coefficient as illustrated in Table 2. These thresholds were chosen experimentally, by visually inspecting a set of signal samples associated with different correlation values.

**Table 2:** Pearson correlation categories between window of patient-acquired signal and reference signal

Coefficient *R*	Correlation group
$R \ge 0.8$	Group I
$0.6 \le R < 0.8$	Group II
$0.5 \le R < 0.6$	Group III
$R < 0.5$	Group IV

**Fig. 3:**
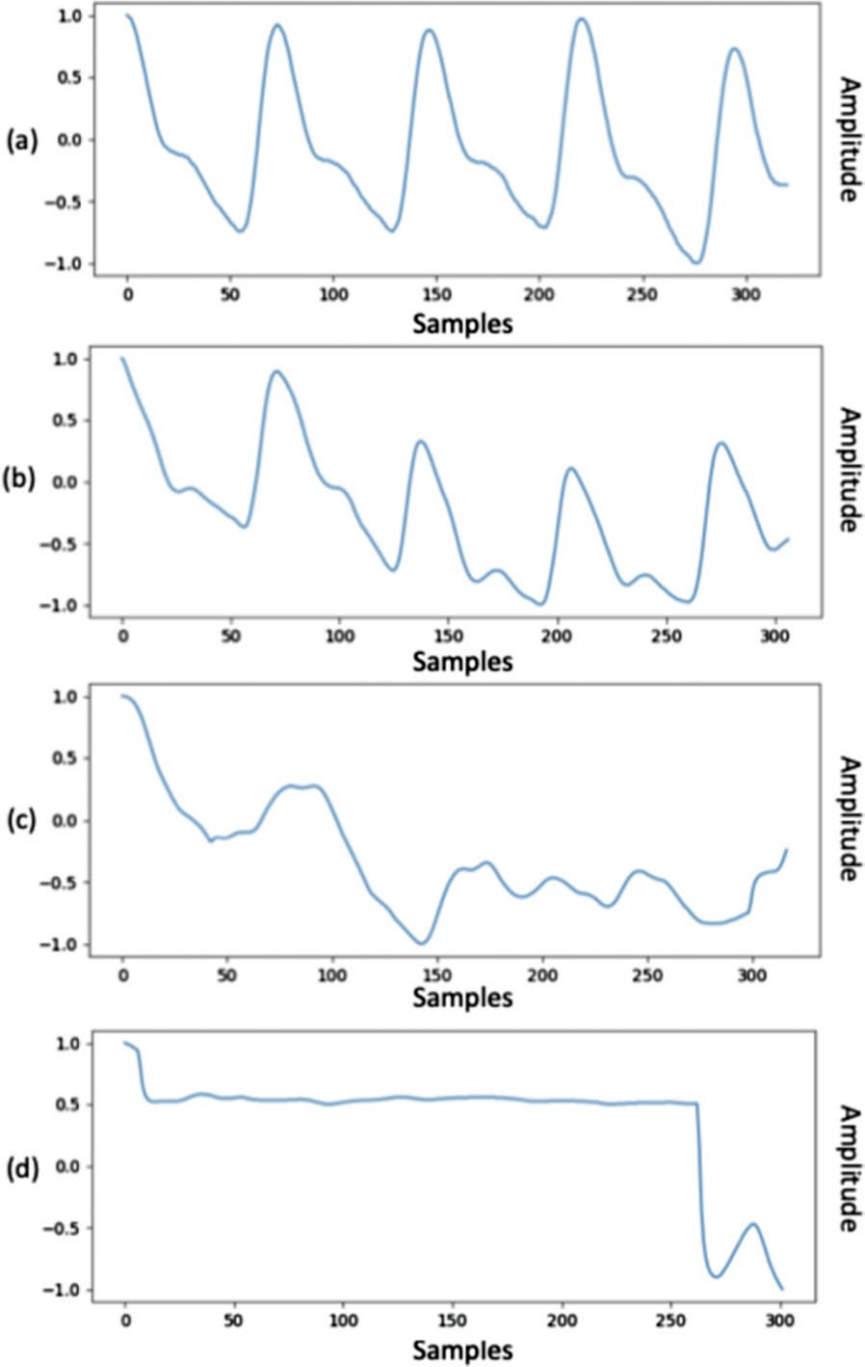
Examples of classified 3-s windows according to Pearson’s correlation coefficient. The figure shows that samples belonging to different groups have a different quality. The signals of group I (**a**) and group II (**b**) present the typical morphology of the PPG signal. The signals of group III (**c**) and group IV (**d**), associated with lower values of the correlation coefficient, are of poor quality

An example of samples from each correlation group is shown in Fig. 3.

Segments containing windows belonging to group III or IV (Pearson correlation coefficient lower than 0.6) were discarded.

As a result, we obtained 720 h of recording from 139 control subjects and 2272 h of recording from 111 sepsis subjects.

Finally, the training and test sets were created. The subjects were randomly assigned to training and test sets using 80% and 20% of ratio, respectively. Segments from a single patient could not appear in both sets.

Moreover, the maximum amount of data per patient was set to 3 h in order to avoid a patient being over-represented. For patients with available signal greater than 3 h, the segments were selected by random sampling. After the data selection phase, some patients were represented only by a few data samples. We considered that a small number of samples could indicate an unreliable signal with a high signal-to-noise ratio. Therefore, we set a minimum threshold of 1 h of signal per patient in order to further improve the quality of the data set. Patients who did not have the required amount of minimal signal were excluded. A statistical description of the resulting training set and the test set is shown in Table 3.

**Table 3:** Data set description

	Training set	Test set
Control group		
No. samples	6600	1598
No. subjects	81	20
Ratio (%)	53.2%	53.7%
Sepsis group		
No. samples	5816	1377
No. subjects	68	17
Ratio (%)	46.8%	46.3%
Total samples	12,416	2975
Length (h)	413 h	99 h

**Fig. 4:**
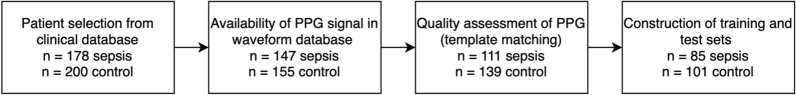
Main steps in dataset construction. For each step, the number of subjects involved is given for the septic group and the control group

After the patient selection process, data from 85 sepsis patients and 101 controls were considered. Figure 4 summarizes the procedure used for defining the dataset.

### Network model

**Fig. 5:**
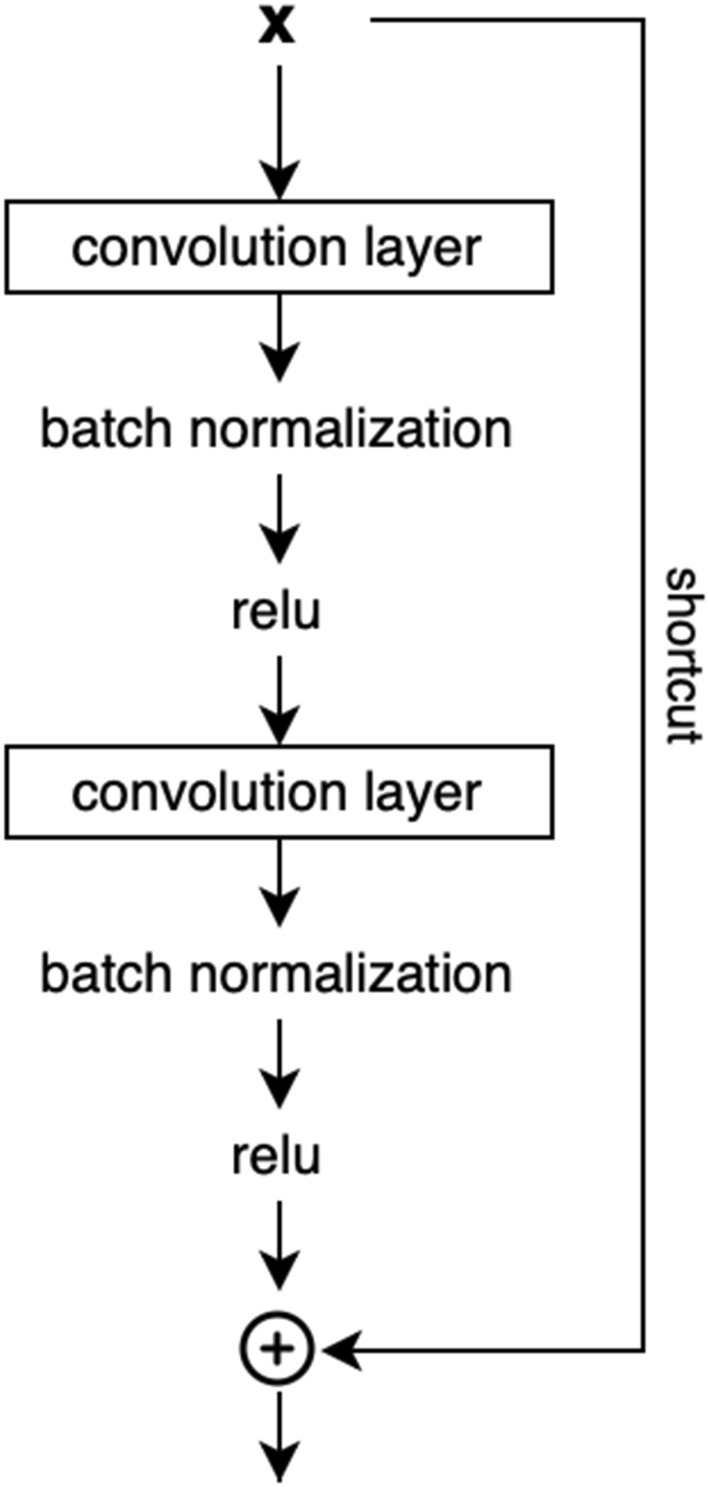
Composition of the identity block. The identity block consists of two convolutional layers, each followed by a batch normalization layer and a ReLU activation. Output of the identity block is created by summing the input to the identity block and the output of the last ReLU activation

We based our model on a widely used ResNet architecture [46]. Our model’s architecture started with an input layer, followed by a single convolutional and a max pooling layer. After this, we added 8 identity blocks separated by max pooling layers. Each identity block, illustrated in Fig. 5, included a shortcut connection and two convolutional layers initialized using Glorot function. Each convolutional layer was followed by a batch normalization layer, and ReLU activation. The shortcut connection performed sum of the input to the identity block and the output of the last ReLU activation.

**Fig. 6:**
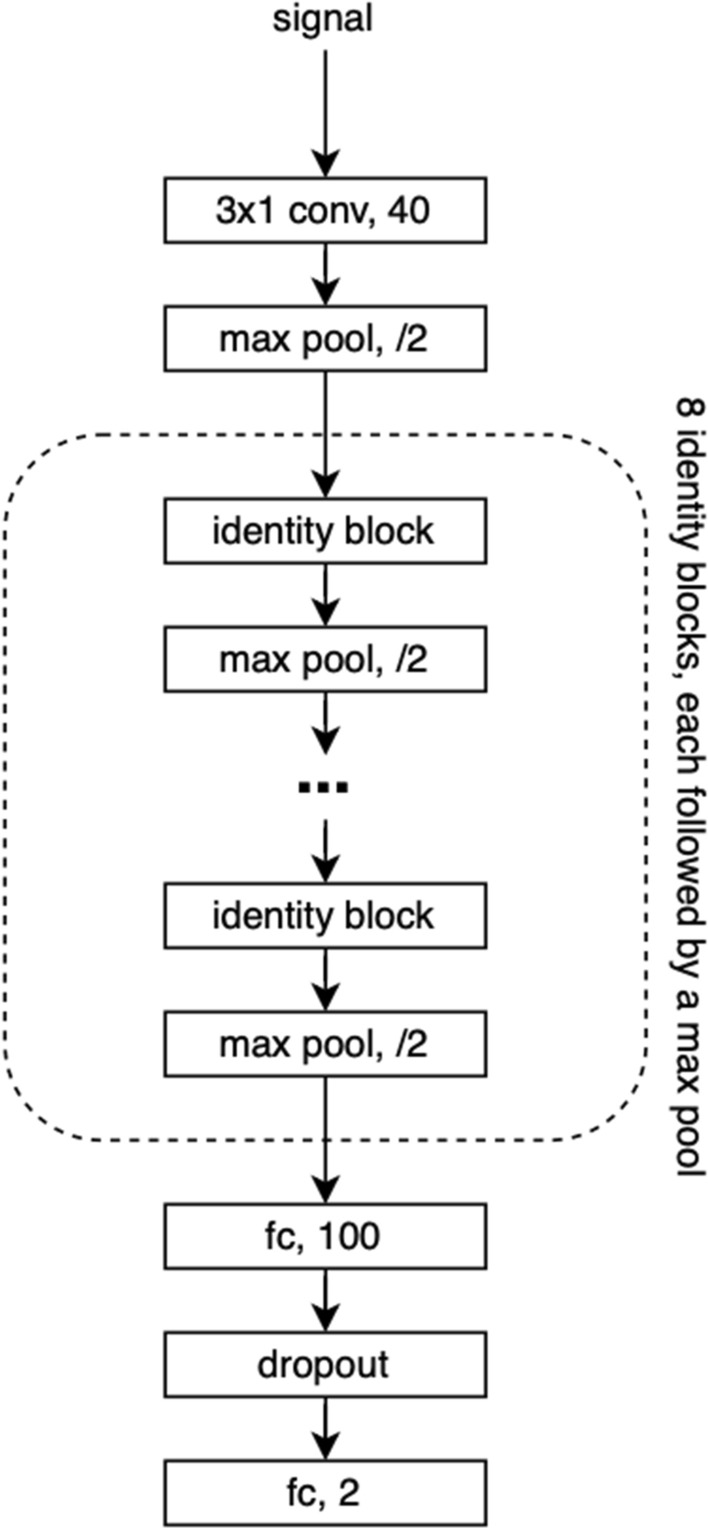
The network architecture used in our study. The core of the network consists of 8 consecutive identity blocks and max pooling layers. At the end of the network two fully connected layers separated by a dropout layer are present

After the identity blocks, we added a fully connected dense layer with 100 units, a dropout layer with 0.2 dropout rate, and lastly, a fully connected layer with a Softmax activation. Dense layers used the same initialization function as the convolutional layers. As an input, the model used raw 2 minutes PPG segments, normalized within the range [$-1$, 1]. All convolutional layers had a number of filters equal to 40, with a filter width equal to 3. A depiction of the complete architecture is illustrated in Fig. 6.

### Evaluation

To evaluate the performance, we trained the model using k-fold cross-validation. The k-fold cross-validation involves dividing the training data into approximately equal size k subsets, called “Folds”. The model training is then repeated iteratively k times, so that at each iteration one of the folds is used as the validation set and the other $k-1$ folds constitute the training data. In this work, the k value was chosen to be 5, resulting the training data to be split into 5 separate folds. The division of the data was done by ensuring that PPG samples acquired from the same subject were not present in multiple folds. As a result of this approach we obtained 5 different models, each of which was trained and validated on data from different patients. The best model weights from each training iteration were selected using validation loss as a metric. These weights were then used as an ensemble to perform majority voting prediction on the test set.

### Parameter optimization

To train the model, we set the learning rate to 1e−6, batch size to 128, and number of epochs to 800. In addition, we used Adam optimizer and binary cross-entropy loss.

To select the optimal parameters, we conducted several experiments that led to the final version of the model. In this section we discuss the selection of the architecture, hyperparameters, input data length and data presentation format.

#### Architecture

We chose ResNet architecture [46] for this project due to its prominent status and signal classification capabilities demonstrated by literature [47]. Once the architecture type was chosen, we ran several empirical experiments in order to determine suitable depth for the network. Based on the achieved results we chose architecture depth consisting of 8 identity blocks.

#### Learning rate

One of the most important hyperparameters is learning rate which typically has values ranging between less than 1 and 1e−6 [48]. Learning rate defines how large updates are applied to the model weights during backward pass in response to the estimated error. In our study, we found experimentally a suitable learning rate by running multiple training with various, commonly used, learning rates. Learning rates of negative powers of 10 ranging from 1e−2 to 1e−7 were evaluated. When deciding on appropriate value, we considered quantitative metrics such as maximum accuracy, minimum loss, and qualitative metrics such as perceived smoothness of the learning, convergence and absence of under- or overfitting. Based on these metrics, we chose learning rate of 1e−6.

#### Batch size

Smaller batch sizes have been shown to improve generalization [49], but they can be computationally less effective than larger batches [48]. In this study, we performed experiments using batch sizes of power of 2, ranging from 16 to 1024. Based on the experiments, we chose batch size of 128, which resulted in a good balance between computational efficiency and accuracy.

#### Data augmentations

Deep learning thrives on large datasets, but often available training data is scarce. In order to reduce this issue, data augmentations is commonly use to increase amount of training data. However, in the case of biosignals, the design of data augmentation techniques needs to consider that it is necessary to preserve the time domain characteristics that represent physiological phenomena [50].

In this study, we evaluated effectiveness of adding noise and using random windows. The noise was sampled from a normal distribution and added to the normalized PPG signal. After noise addition, the resulting noisy signal was normalized again to obtain the values within the [$-1$, 1] range expected by the model. The noise augmentation was applied to the signal with 50% of chance. Random windows were implemented by taking a continuous 90 s window from a random location of the 2 min PPG segment. As shown in Table 4, the use of data augmentations did not lead to a significant improvement in the performance. The combined use of jitter and windows led to modest improvements in accuracy and specificity compared to the model without augmentations. As the baseline approach yielded the best performance on sensitivity, it was selected for the final version of the model.

**Table 4:** Augmentation results

Model	Accuracy (%)	Sensitivity (%)	Specificity (%)
Baseline	76.37	70.95	81.04
Jitter	75.70	68.34	82.04
Windows	73.71	65.50	80.79
Jitter & Windows	76.47	69.43	82.54

#### Segment length

Moreover, we explored classification of photoplethysmography segments of various lengths. Exploration started with segments lasting 1 h. Subsequently, the length of the segments considered was gradually reduced to 1 min. Shorter segments increased amount of data and led to improved signal-to-noise ratio as shorter segments allowed visual inspection of the signal, which helped in identifying and discarding various artifacts. Furthermore, shorter segments allowed assessing the effectiveness of signal quality metrics.

#### Frequency domain presentation

We investigated frequency domain presentation input in addition to raw time-series. We observed a trend where frequency domain presentation compared favorably to the time-series when the PPG segments were longer, but when the segments were shorter, frequency presentation lost its advantage. We hypothesized that the better outcome obtained with the longer segments might have been due to simplified presentation in the frequency domain.

## Results

All trained models and their ensemble, with corresponding evaluation metrics, are shown in Table 5. Each model is indicated in the table by the name of the fold used as the validation set. Accuracy identifies the percentage of correctly classified samples. Sensitivity indicates proportion of correctly classified sepsis samples, and specificity shows the percentage of correctly predicted control samples.

**Fig. 7:**
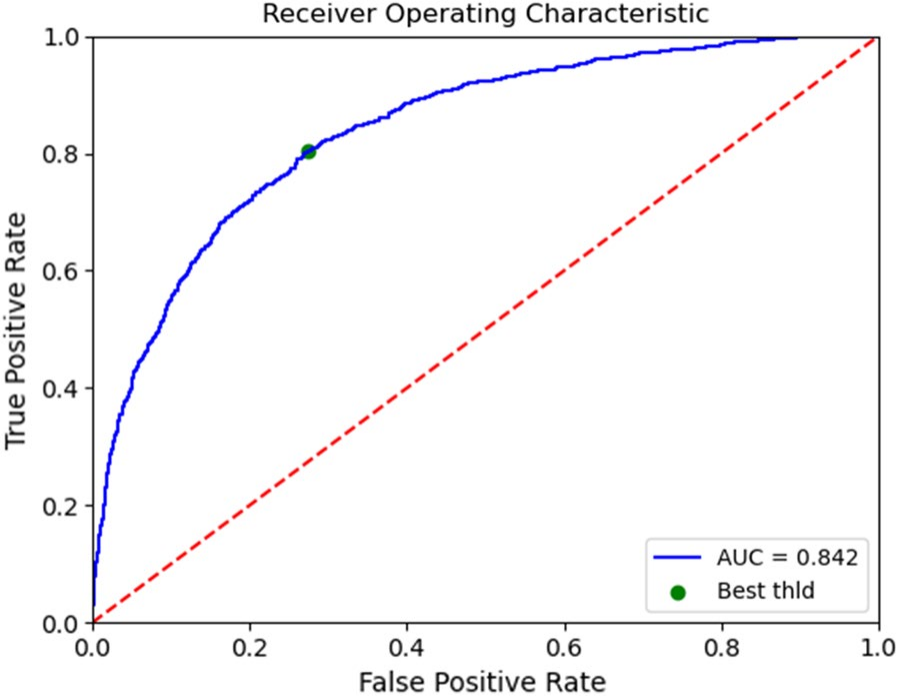
Calculated ROC curve. Red diagonal line represents points where the true positive rate is equal to the false positive rate. Points to the left of the diagonal line mean that proportion of true positives is higher than false positives. Optimal value is at the top of the left corner

As shown in Table 5, the accuracy between the models varies from 72.27 to 74.59%, sensitivity from 65.72 to 71.31% and specificity from 76.47 to 79.72%. The ensemble method achieves 76.37% of accuracy with sensitivity of 70.95% and specificity of 81.04%. In addition to accuracy, sensitivity and specificity, Receiver Operating Characteristic (ROC) curve was calculated for the ensemble. The ROC curve, illustrated in Fig. 7, shows that our method reaches 0.842 of Area Under Curve (AUC).

**Table 5:** Evaluation results

Model	Accuracy (%)	Sensitivity (%)	Specificity (%)
Fold 0	72.87	65.72	79.04
Fold 1	73.65	67.83	78.66
Fold 2	73.61	66.52	79.72
Fold 3	74.59	71.31	77.41
Fold 4	72.27	67.39	76.47
Ensemble	76.37	70.95	81.04

**Fig. 8:**
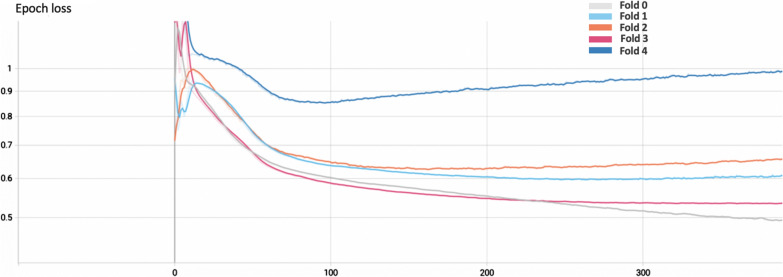
Validation loss curves for the first 400 training epochs. In the figure, folds 0 and 3 have not yet reached plateau, in contrast to folds 2 and 4, which show a trend that could indicate overfitting on the training data. Lower loss values indicate better performance

Figure 8 shows the trend of the loss function on the validation set for all 5 models during the first 400 training epochs. The loss curves show that the model identified as Fold 0 (gray) achieves the lowest loss, whereas Fold 4 (blue) performs the worst.

## Discussion

This study allowed investigating the feasibility of using deep learning based method to classify sepsis through the only analysis of photoplethysmogram signal. In particular, we developed a deep learning based model and trained it on PPG signal extracted from the public ICU waveform database (MIMIC-III database). To the best of our knowledge, this is the first study aimed at verifying the possibility of using only photoplethysmogram signal together with a deep learning based method to classify sepsis. As regards data analysis, 5-fold cross-validation was used, which resulted in 5 models, each trained using different training and validation subjects. Due to the differences in the training and validation data in each cross-validation iteration, the performances of the models were different when evaluated on the test set. Our method showed mean and standard deviation of $73.398 \pm 0.784$ for accuracy, $67.754 \pm 1.921$ for sensitivity and $78.26 \pm 1.168$ for specificity. In this regard, it was hypothesized that the performance variations between folds depended on how well the training-validation data split of a given fold represented the test data; this also suggest the selected dataset contains not homogeneous populations, thus a larger dataset may improve the classification results. The final prediction was carried out by using majority-voting which consulted all the trained models. Due to the differences among the folds, the ensemble method was presumed to make a better decision than any of the models independently. By using the ensemble, we achieved 76.37% of accuracy, 70.95% of sensitivity and 81.04% of specificity, demonstrating promising results and indicating the possibility to use a PPG signal to assist in diagnosing sepsis. Our method could provide support to the sepsis diagnostic process, and allow a more timely diagnosis. Importantly, the method might not require recording extra signals because PPG is already commonly recorded in the case of ICU patients. Moreover, the acquisition and processing of photoplethysmogram signals can ensure continuous low-cost monitoring of the patient with or at risk of sepsis.

Differently from previous studies, the model proposed in this article performs a binary classification of sepsis and was trained using only the raw plethysmographic signal. Previous studies using the MIMIC database for sepsis detection were mainly conducted using vital parameters and laboratory measurements. Among these, we feel it is worth mentioning some works that used a deep learning approach for sepsis identification.

**Table 6:** Summary of the results of other works on sepsis identification from MIMIC database; abbrevations: Accuracy (ACC), Sensitivity (SE), Specificity (SP)

Authors	Method	Prediction time	Input parameters	AUC	ACC	SE	SP
Calvert et al.	Insight machine learning algorithm	3 h before estimated onset	Systolic blood pressure, pulse pressure, heart rate, body temperature, respiration rate, white blood cell count, pH, blood oxygen saturation, age	0.92	82.70%	90.00%	81.00%
Desautels et al.	Insight machine learning algorithm	4h before estimated onset	Age, systolic blood pressure, pulse pressure, heart rate, respiration rate, Sp02, Glasgow Coma Score	0.74	–	80.00%	54.00%
Mollura et al.	Bagged Tree Classifier	1 h from ICU admission	Features extracted from ECG and ABP waveforms	0.86	85.00%	85.00%	86.00%
Kam et al.	LSTM (Long-short term memory network)	3h before estimated onset	Systolic Blood Pressure, Pulse Pressure, Heart Rate, Body Temperature, Respiration Rate, White Blood Cell Count, pH, Blood Oxygen Saturation, Age	0.93	93.00%	91.40%	94.40%
Ashuroğlu et al.	CNN (Convolutional Neural Network) and Random Forest	6h before estimated onset	Heart Rate, Systolic and Diastolic Blood Pressure, Respiratory Rate, Oxygen Saturation, Glasgow Coma Score eye opening, Temperature	0.97	–	–	–
Scherpf et al.	RNN (Recurrent Neural Network)	3h before estimated onset	Systolic and Diastolic Blood Pressure, Heart Rate, Body Temperature, Respiration Rate, White Blood Cell Count, pH, Blood Oxygen Saturation, Age	0.81	–	90.00% *	46.90%
Present Method	Custom ResNet	0h	PPG raw timeseries	0.84	76.37%	70.95%	81.04%

*For the calculation of the specificity the sensitivity was fixed to 90.00%

Kam and Kim [51] extracted the minimum, mean and maximum values of hourly periods of vital signs and laboratory measurement parameters from the MIMIC-II database. The extracted features were used to train different architectures to predict sepsis 3 h before the estimated onset time. The authors reported that the Long Short-Term Memory (LSTM) architecture was the most effective, based on the Area Under the Curve (AUC) criterion.

Ashuroğlu et al. [52] proposed a model called Deep SOFA-Sepsis Prediction Algorithm, which combined CNN and Random Forest algorithms, to predict the SOFA score. The authors trained the model with 7 vital signs obtained from MIMIC-III database. Laboratory results were excluded in order to assess the feasibility of estimating the risk score. They evaluated the architecture of their performance also to predict sepsis 6 h before the estimated onset time.

Scherpf et al. [53] developed a Recurrent Neural Network (RNN) model to predict sepsis 3 h before the estimated onset time. The model was trained using white blood cell count and vital signs averaged over one-hour intervals. The training data were obtained from MIMIC-III database.

Our method achieved AUC of 0.842 compared to 0.929 reported by Kam and Kim [51], 0.972 by Ashuroğlu [52], and 0.81 by Scherpf [53].

Table 6 summarises in more detail the cited works that used the MIMIC database for the identification of sepsis. Although these studies performed better than our method, we believe the results we obtained can still be considered very interesting, as our method only uses the PPG signal as input.

Our study has some limitations. As reported in the MIMIC-III database documentation, the ICD-code was generated at the end of the hospitalisation, consequently information on when the diagnosis was made or when the patient showed the symptoms is not known. Due to this limitation, our subject selection consisted of those sepsis patients who were hospitalised only once in ICU. We hypothesised that by using this criterion, the corresponding signals contained sufficient information on the target pathology.

Nevertheless, it should be mentioned that some studies have tried to estimate the onset time of sepsis in the MIMIC database on the basis of the diagnostic criteria for sepsis: presence of 2 or more SIRS criteria or SOFA score $>2$. After extracting the parameters necessary to estimate the SIRS or the SOFA scores, several authors [26, 27, 52, 53] considered the onset time of the disease to be when the estimated score met the diagnostic criterion for sepsis. Second limitation of the study is the selection of control and target diagnoses. In our study, the control group was restricted to a small subset (n = 5) of ICD-9 mental disorders, and sepsis group consisted of multiple (n = 3) classes of different sepsis severities. Furthermore, all waveforms used for training and testing the method were collected from the same database.

Based on the above limitations, the future direction of this research involves evaluating the model on a larger set of control diagnoses as well as sepsis diagnoses stratified by severity. Patient selection could be improved by including subjects from other databases and by extending the subject selection in MIMIC-III. To assess the generalisation capability of our model, we intend to test the performance of our method on other datasets. Furthermore, to evaluate the ability of the method in predicting the onset of sepsis, we plan to train and test the model on a dataset where the diagnosis times are known.

## Conclusion

This study explored the feasibility of using a deep learning based method to classify sepsis relying only on the photoplethysmogram signal. This was possible through the use and analysis of the MIMIC-III database. The proposed method allowed us to achieve AUC of 0.842 and obtain an accuracy of 76.37% on the testing set demonstrating promising results. The proposed method, using only a non-invasive signal, is perfectly suited for long-term monitoring of patients at risk. We hypothesize this method could serve as an early warning system to trigger application of more invasive tests, and thus reduce the time to make a diagnosis. This method could contribute in improving the quality of the treatment of patients. However, as discussed in Chapter 4, future studies with a larger number of patients and data from other databases will be necessary to assess the effectiveness of the proposed method.

## References

[1] Singer M, Deutschman C, Seymour C, Shankar-Hari M, Annane D, Bauer M, Bellomo R, Bernard G, Chiche J-D, Coopersmith C, Hotchkiss R, Levy M, Marshall J, Martin G, Opal S, Rubenfeld G, Poll T, Vincent J-L, Angus D (2016). The third international consensus definitions for sepsis and septic shock (sepsis-3). JAMA.

[2] Rudd K, Johnson S, Agesa K, Shackelford K, Tsoi D, Kievlan D, Colombara D, Ikuta K, Kissoon N, Finfer S, Fleischmann C, Machado F, Reinhart K, Rowan K, Seymour C, Watson S, West E, Marinho de Souza MDF, Hay S, Naghavi M (2020). Global, regional, and national sepsis incidence and mortality, 1990–2017: analysis for the global burden of disease study. Lancet.

[3] Sakr Y, Jaschinski U, Wittebole X, Szakmany T, Lipman J, Namendys-Silva S, Martin-Loeches I, Leone M, Lupu M, Vincent J-L (2018). Sepsis in intensive care unit patients: Worldwide data from the icon audit. Open Forum Infect Dis.

[4] Dugar S, Choudhary C, Duggal A (2020). Sepsis and septic shock: guideline-based management. Clevel Clin J Med.

[5] Ramdeen S, Ferrell B, Bonk C, Schubel L, Littlejohn R, Capan M, Arnold R, Miller K (2021). The available criteria for different sepsis scoring systems in the emergency department-a retrospective assessment. Open Access Emerg Med OAEM.

[6] Kumar A, Roberts D, Wood K, Light B, Parrillo J, Sharma S, Suppes R, Feinstein D, Zanotti S, Taiberg L, Gurka D, Kumar A, Cheang M (2006). Duration of hypotension before initiation of effective antimicrobial therapy is the critical determinant of survival in human septic shock. Crit Care Med.

[7] Evans L, Rhodes A, Alhazzani W, Antonelli M, Coopersmith CM, French C, Machado FR, Mcintyre L, Ostermann M, Prescott HC (2021). Surviving sepsis campaign: international guidelines for management of sepsis and septic shock 2021. Intensive Care Med.

[8] Ferrer R, Martin-Loeches I, Phillips G, Osborn T, Townsend S, Dellinger R, Artigas A, Schorr C, Levy M (2014). Empiric antibiotic treatment reduces mortality in severe sepsis and septic shock from the first hour. Crit Care Med.

[9] Marik PE (2014). Don’t miss the diagnosis of sepsis!. Crit Care.

[10] Spoto S, Nobile E, Carnà EPR, Fogolari M, Caputo D, De Florio L, Valeriani E, Benvenuto D, Costantino S, Ciccozzi M (2020). Best diagnostic accuracy of sepsis combining sirs criteria or GSOFA score with procalcitonin and mid-regional pro-adrenomedullin outside ICU. Sci Rep.

[11] Rangel-Frausto MS, Pittet D, Costigan M, Hwang T, Davis CS, Wenzel RP (1995). The natural history of the systemic inflammatory response syndrome (SIRS): a prospective study. JAMA.

[12] Vincent J-L, Moreno R, Takala J, Willatts S, De Mendonça A, Bruining H, Reinhart C, Suter P, Thijs LG (1996). The SOFA (Sepsis-related Organ Failure Assessment) score to describe organ dysfunction/failure. Springer.

[13] Zhang Z, Smischney NJ, Zhang H, Van Poucke S, Tsirigotis P, Rello J, Honore PM, Kuan WS, Ray JJ, Zhou J (2016). Ame evidence series 001-the society for translational medicine: clinical practice guidelines for diagnosis and early identification of sepsis in the hospital. J Thorac Dis.

[14] Mignot-Evers L, Raaijmakers V, Buunk G, Brouns S, Romano L, van Herpt T, Gharbharan A, Dieleman J, Haak H (2021). Comparison of SIRS criteria and GSOFA score for identifying culture-positive sepsis in the emergency department: a prospective cross-sectional multicentre study. BMJ Open.

[15] Brunetti E, Isaia G, Rinaldi G, Brambati T, De Vito D, Ronco G, Bo M (2021). Comparison of diagnostic accuracies of GSOFA, news, and mews to identify sepsis in older inpatients with suspected infection. J Am Med Dir Assoc.

[16] Charlton M, Sims M, Coats T, Thompson JP (2017). The microcirculation and its measurement in sepsis. J Intensive Care Soc.

[17] De Backer D, Donadello K, Sakr Y, Ospina-Tascon G, Salgado D, Scolletta S, Vincent J-L (2013). Microcirculatory alterations in patients with severe sepsis: impact of time of assessment and relationship with outcome. Crit Care Med.

[18] Ait-Oufella H, Lemoinne S, Boelle P, Galbois A, Baudel J, Lemant J, Joffre J, Margetis D, Guidet B, Maury E (2011). Mottling score predicts survival in septic shock. Intensive Care Med.

[19] Ait-Oufella H, Joffre J, Boelle P, Galbois A, Bourcier S, Baudel J, Margetis D, Alves M, Offenstadt G, Guidet B (2012). Knee area tissue oxygen saturation is predictive of 14-day mortality in septic shock. Intensive Care Med.

[20] Coudroy R, Jamet A, Frat J-P, Veinstein A, Chatellier D, Goudet V, Cabasson S, Thille AW, Robert R (2015). Incidence and impact of skin mottling over the knee and its duration on outcome in critically ill patients. Intensive Care Med.

[21] Sorelli M, Francia P, Bocchi L, De Bellis A, Anichini R (2019). Assessment of cutaneous microcirculation by laser doppler flowmetry in type 1 diabetes. Microvasc Res.

[22] Bandini A, Orlandi S, Manfredi C, Evangelisti A, Barrella M, Bevilacqua M, Bocchi L (2013). Effect of local blood flow in thermal regulation in diabetic patient. Microvasc Res.

[23] Sorelli M, Stoyneva Z, Mizeva I, Bocchi L (2017). Spatial heterogeneity in the time and frequency properties of skin perfusion. Physiol Meas.

[24] Piepoli M, Garrard CS, Kontoyannis D, Bernardi L (1995). Autonomic control of the heart and peripheral vessels in human septic shock. Intensive Care Med.

[25] Middleton PM, Tang CH, Chan GS, Bishop S, Savkin AV, Lovell NH (2011). Peripheral photoplethysmography variability analysis of sepsis patients. Med Biol Eng Comput.

[26] Calvert JS, Price DA, Chettipally UK, Barton CW, Feldman MD, Hoffman JL, Jay M, Das R (2016). A computational approach to early sepsis detection. Comput Biol Med.

[27] Desautels T, Calvert J, Hoffman J, Jay M, Kerem Y, Shieh L, Shimabukuro D, Chettipally U, Feldman MD, Barton C (2016). Prediction of sepsis in the intensive care unit with minimal electronic health record data: a machine learning approach. JMIR Med Inform.

[28] Mao Q, Jay M, Hoffman JL, Calvert J, Barton C, Shimabukuro D, Shieh L, Chettipally U, Fletcher G, Kerem Y (2018). Multicentre validation of a sepsis prediction algorithm using only vital sign data in the emergency department, general ward and icu. BMJ Open.

[29] Mollura M, Mantoan G, Romano S, Lehman L-W, Mark RG, Barbieri R. The role of waveform monitoring in sepsis identification within the first hour of intensive care unit stay. In: 2020 11th Conference of the European Study Group on Cardiovascular Oscillations (ESGCO), pp. 1–2 (2020). 10.1109/ESGCO49734.2020.9158013

[30] Rim B, Sung N-J, Min S, Hong M (2020). Deep learning in physiological signal data: a survey. Sensors.

[31] Ganapathy N, Swaminathan R, Deserno TM (2018). Deep learning on 1-d biosignals: a taxonomy-based survey. Yearb Med Inform.

[32] Miotto R, Wang F, Wang S, Jiang X (2017). Deep learning for healthcare: review, opportunities and challenges. Brief Bioinform.

[33] Kiranyaz S, Ince T, Abdeljaber O, Avci O, Gabbouj M. 1-d convolutional neural networks for signal processing applications. In: ICASSP 2019-2019 IEEE International Conference on Acoustics, Speech and Signal Processing (ICASSP), pp. 8360–8364 (2019). 10.1109/ICASSP.2019.8682194.

[34] Alaskar H (2018). Convolutional neural network application in biomedical signals. J Comput Sci Inform Tech.

[35] Schlesinger O, Vigderhouse N, Eytan D, Moshe Y. Blood pressure estimation from ppg signals using convolutional neural networks and siamese network. In: ICASSP 2020-2020 IEEE International Conference on Acoustics, Speech and Signal Processing (ICASSP), pp. 1135–1139 (2020). 10.1109/ICASSP40776.2020.9053446.

[36] Liang Y, Chen Z, Ward R, Elgendi M (2018). Photoplethysmography and deep learning: enhancing hypertension risk stratification. Biosensors.

[37] Johnson AE, Pollard TJ, Shen L, Lehman LH, Feng M, Ghassemi M, Moody B, Szolovits P, Anthony Celi L, Mark RG (2016). MIMIC-III, a freely accessible critical care database. Sci Data.

[38] Moody B, Moody G, Villarroel M, Clifford G, Silva I (2020). MIMIC-III waveform database (version 1.0). PhysioNet.

[39] Moody B, Craig M, Johnson A, Kyaw T, Moody G, Saeed M, Villarroel M. The MIMIC-III waveform database matched subset, physionet. org. Physionet (2020). 10.13026/c2294b

[40] Lombardi S, Partanen P, Bocchi L. Detecting sepsis from photoplethysmography: strategies for dataset preparation. In: Proceedings of the IEEE Conference (2022). 10.1109/EMBC48229.2022.9871973 10.1109/EMBC48229.2022.9871973 36086115

[41] Xie C, McCullum L, Johnson A, Pollard T, Gow B, Moody B (2021). Waveform database software package (WFDB) for python (version 3.3.0). PhysioNet.

[42] Sukor JA, Redmond S, Lovell N (2011). Signal quality measures for pulse oximetry through waveform morphology analysis. Physiol Meas.

[43] Orphanidou C, Bonnici T, Charlton P, Clifton D, Vallance D, Tarassenko L (2014). Signal-quality indices for the electrocardiogram and photoplethysmogram: derivation and applications to wireless monitoring. IEEE J Biomed Health Inform.

[44] Makowski D, Pham T, Lau ZJ, Brammer JC, Lespinasse F, Pham H, Schölzel C, Chen SA (2021). Neurokit2: a python toolbox for neurophysiological signal processing. Behav Res Methods.

[45] Elgendi M, Norton I, Brearley M, Abbott D, Schuurmans D (2013). Systolic peak detection in acceleration photoplethysmograms measured from emergency responders in tropical conditions. PLoS ONE.

[46] He K, Zhang X, Ren S, Sun J. Deep residual learning for image recognition. In: Proceedings of the IEEE Conference on Computer Vision and Pattern Recognition, pp. 770–778 (2016). 10.1109/CVPR.2016.90

[47] O’shea TJ, Roy T, Clancy TC (2018). Over-the-air deep learning based radio signal classification. IEEE J Select Top Signal Process.

[48] Bengio Y. Practical recommendations for gradient-based training of deep architectures. In: Neural Networks: Tricks of the Trade, pp. 437–478. Springer, Heidelberg (2012). 10.1007/978-3-642-35289-8_26

[49] Shirish Keskar N, Mudigere D, Nocedal J, Smelyanskiy M, Tang PTP. On large-batch training for deep learning: generalization gap and sharp minima. 1609 (2016)

[50] Lashgari E, Liang D, Maoz U (2020). Data augmentation for deep-learning-based electroencephalography. J Neurosci Methods.

[51] Kam HJ, Kim HY (2017). Learning representations for the early detection of sepsis with deep neural networks. Comput Biol Med.

[52] Aşuroğlu T, Oğul H (2021). A deep learning approach for sepsis monitoring via severity score estimation. Comput Methods Program Biomed.

[53] Scherpf M, Gräßer F, Malberg H, Zaunseder S (2019). Predicting sepsis with a recurrent neural network using the mimic iii database. Comput Biol Med.

